# Formal Quality and Compliance of Informed Consent Forms in Critical Care and Surgical Areas in Spain: An Observational Study

**DOI:** 10.3390/nursrep13010004

**Published:** 2022-12-31

**Authors:** José Manuel García-Álvarez, José Luis Díaz-Agea, María Suárez-Cortés, Alonso Molina-Rodríguez, Ismael Jiménez-Ruiz, Alfonso García-Sánchez

**Affiliations:** 1 Health Sciences PhD Program, Universidad Católica de Murcia UCAM, Campus de los Jerónimos nº135, Guadalupe, 30107 Murcia, Spain; 2 Faculty of Nursing, University of Murcia, Campus de Ciencias de la Salud, Edificio LAIB/DEPARTAMENTAL, El Palmar-Murcia, 30120 Murcia, Spain; 3 Faculty of Nursing, Catholic University of Murcia, Guadalupe, 30107 Murcia, Spain

**Keywords:** informed consent, patient’s freedom to choose, compliance, critical care, surgical areas, consent forms, nursing professionals

## Abstract

(1) Background: The informed consent form must contain all the relevant information about the procedure to be performed to guarantee the patient’s freedom to choose. (2) Objective: To analyze the formal quality of, and compliance with informed consent forms in critical care and surgical areas in a county hospital in Spain. (3) Methods: The formal quality of informed consent forms in critical care and surgical areas from the hospital were analyzed, following the established formal quality criteria for informed consent forms. The compliance with specific criteria for each of the operated patients during the period of study was also evaluated. (4) Results: The formal quality of 224 informed consent forms was analyzed from 8 disciplines observing a median of non-compliances of 4 with a minimum of 1 and a maximum of 5, with the most breaches being in verifying the delivery of a copy to the patient and showing contraindications. The compliance of 376 documents from 188 operated patients were assessed, highlighting that the non-complied items were: the personalized risks and complete identification of the patient and the physician. A significant association was found between disciplines analyzed and the identification of the physician and personalized risks, with anesthesia and critical care showing the best compliance. (4) Conclusions: The informed consent forms in critical care and surgical areas were shown to have a deficient formal quality and an inadequate compliance. These deficiencies should be corrected to improve the information received by the patients and to guarantee their freedom to choose. As nurses have a responsibility to ensure that patients are adequately informed about both nursing interventions and care, as well as the surgical treatments they receive, consideration should be given to the possibility of nursing professionals taking the lead in obtaining informed consent.

## 1. Introduction

Continuing technological advances in health sciences have made necessary the development of a bridge that brings together different aspects in the relationship between healthcare professionals and patients: scientific, judicial, ethical, and personal aspects. One part of the bridge is the promotion of patient empowerment through information to improve decision-making in health services [1]. This task mainly falls to the process of informed consent, which guarantees the right of every patient to information. It allows the patient to freely, actively, and voluntarily participate in any procedure that influences the patient’s health [2,3,4,5,6].

The informed consent form must be an ethical and legal document that is: specific for each treatment, allows the patient to accept or refuse treatment or diagnostic tests in accordance with their values and interests, within a process of dialogue, and developed within the framework of the professional-patient relationship [7]. Therefore, this document is a tool for transmitting information which should include understandable, important, and relevant information that the patient needs to know before making a significant decision [8]. This information includes description, aim, benefits, relevant consequences, probable or typical risks of the procedure, personalized risks as a function of the patient’s characteristics, contraindications, and alternatives. All information should be based on available empirical knowledge. Also, a section where the patient can revoke this document at any time must be present [9,10,11].

The lack of some of these pieces of information, or the lack of adequate compliance, invalidates the document from a legal and ethical point of view, as it does not provide the patient with all the necessary information for making an informed decision. For this, compliance should be specific for each patient and should be adapted to the patient’s own characteristics. This will guarantee not only that the patient is aware about all the general aspects of the procedure he or she will be subjected to, but also the specific implications that this procedure could have for the patient [12,13,14]. For example, when the information provided to the patient is effective and complete, pre-surgery stress, fear and anxiety can be reduced in some cases [1].

The patient’s consent could be oral, and it is preferable that it is also found in writing in the informed consent form [15,16,17,18,19]. In Spain, in actual clinical practice, informed consent is generally obtained orally, and written consent is the exception; the procedures in which informed consent must be obtained in written form are set out in law 41/2022. Although all professionals have responsibilities related to obtaining informed consent, in the clinical setting it is often reduced to an obligation of medical staff. However, nurses have a responsibility to ensure that patients are adequately informed about both nursing interventions and care, as well as the surgical treatments they receive [1,20]. Therefore, informed consent is sometimes obtained by nursing professionals and on other occasions by physicians [21,22], as there are no specific protocols on how it should be obtained, which means that there are sometimes serious deficiencies in obtaining informed consent, sometimes even leaving the patient to read the information alone and, depending on what the patient understands, accept, or reject the proposed intervention [1]. Leaving patients to read the consent form on their own prevents them from asking questions, expressing doubts or concerns, making it a formal act devoid of accompaniment and information.

The objective of the present study was to analyze the formal quality and the compliance with informed consent forms in critical care and surgical areas in a county hospital in Spain.

## 2. Materials and Methods

A quantitative, descriptive, and cross-sectional study was conducted with patients from critical care and surgical areas subjected to a surgical intervention at the “Vega Lorenzo Guirao” Hospital (Cieza, Spain).

The study population was composed of all the patients who had undergone a surgical intervention in the month of November 2020. A non-probabilistic, convenience sampling was performed, which included every patient from any surgical discipline who had a scheduled surgical intervention. A total of 224 documents were analyzed.

In the first place, the formal quality of the all the surgical informed consent forms from the hospital were analyzed, following the formal quality criteria for informed consent forms [9,10,11,12,13,14,23] (Table 1). The documents were analyzed for compliance with these criteria, assigning a point to all the affirmative responses and zero points to the negative ones, to finally obtain an overall formal quality score for each surgery discipline.

The documents from the following surgery disciplines were analyzed: anesthesia and critical care, general surgery, dermatology, gynecology, ophthalmology, otorhinolaryngology, urology, and traumatology.

Afterwards and based on the formal quality criteria of the informed consent forms [9,10,11,12,13,14,23], a compliance form was created, which included 7 dichotomous (Yes/No) answers that referred to all the criteria that should be specifically complied for each specific patient, adding an eighth which indicated the discipline to which the document belonged (Table 2). The compliance with the different items was assessed by exhaustively analyzing the consent forms of the patients who had been subjected to a surgical intervention during the study period.

To analyze the information obtained, IBM SPSS Statistics for Windows (Version 25.0. Armonk, NY, USA: IBM Corp.) was utilized to calculate the frequencies and percentages for the qualitative variables, as well as the mean and standard deviation for the quantitative variables. To assess the existing associations between the different variables analyzed, which were nominal qualitative variables, the Chi-square, Crammer’s V and lambda statistics tests were performed, with a *p* < 0.05 value used to indicate significance.

The manuscript reporting was adhered to the STROBE guideline in the current study [24].

## 3. Results

The formal quality of the 224 informed consent documents of 8 surgical specialties was evaluated, observing a minimum of non-compliance with the formal quality criteria by specialty of 1 and a maximum of 5 with a median of 4.The most violated criteria: 19 (verifying the delivery of a copy to the patient), in 100% of the consent forms, 13 (showing contraindications) in 80.8%, 3 (identification and the signature of the physician) in 61.2% and 12 (space for personalized risks) in 46.9% of the consent forms (Table 3).

Table 4 shows the frequency and percentage of the 376 informed consent documents of the 188 patients who had a surgical intervention during the period of study. It should be considered that each patient has two consents, one corresponding to anesthesia and the other to the surgical specialty to which the patient will be subjected. Table 5 shows the percentage of completion of the items of the form of the documents analyzed.

After the inferential analysis performed between the different variables in this study, it was observed that there was a lack of a statistically significant association between the surgery discipline and the items of the form related to place and date, identification of the patient, patient’s signature, identification of the representative, and signature of the representative. Instead, a statistically significant association was found between the surgery discipline and the items: physician, physician’s signature, and personalized risk, with *p* < 0.001.

Table 6 shows the analysis results according to discipline, considering these last three items, where it is observed that the dermatology discipline showed the least compliance for the adequate identification of the physician, urology had the most physician’s signatures missing and traumatology least reflected personalized risks. In contrast, anesthesia and critical care was the discipline with the most compliance in these three items.

## 4. Discussion

The formal deficiencies in informed consent forms in critical care and surgical areas call into question patient autonomy and the care provided in critical and/or complex situations. Providing comprehensive information and ensuring consent or withdrawal of procedures should be a routine process of care delivery and not a mere bureaucratic formality. To this end, it is necessary to analyze the deficient criteria during the process detected in the study and to assess alternatives to improve the decision-making process of patients in collaboration with the health professionals involved.

In agreement with other research studies conducted in a similar hospital settings and utilizing the same formal quality criteria for the analysis of surgical informed consent forms [9,10], a lower number of non-compliances was found in the present work, which could be due to the interest of the hospital to try to normalize all the informed consent forms. That these specialties lack of some of these specific criteria is a result of the particular guidelines chosen for study, i.e., those set by the corresponding scientific society.

It was also observed, in agreement with other studies [9,10], that the highest number of non-compliances of the formal quality was found in the delivery of a copy to the patients. This could have prevented them from carefully reading it, to adequately assess the practical details of the procedure, and making impossible their consultation of their families or friends for help in making the decision. The informed consent obtained in a short period of time could make the patient decide under pressure, and he or she may not have accepted the procedure if all the aspects of the procedure had been taken into consideration. Therefore, the non-compliance with this formal criteria highly restricts the patients’ freedom of choice, impeding them from actively participating in their healthcare decisions [25]. Moreover, as a matter of law, criminal doctrine in Spain considers that the information to be provided is inversely related to the urgency of the intervention [26].

Another non-compliance found in the documents evaluated, as in previous studies [10,11,12,27,28], was not including specific contraindications about the procedure, or the personalized risks due to the patient’s peculiarities. These are fundamental aspects which could have a decisive influence on the decision-making process, and whose absence could substantially undermine the informed consent process, even going so far as to legally annul it [26].

In the analysis of adequate compliance with the informed consent forms related to the patient’s specific aspects, it was observed—as in other studies [12,14]—that the item with the least-compliance referred to the inclusion of personalized risks. In part, this may be because this information was missing from the document, so that it would be impossible to comply with these criteria. An explanation could be that health professionals are mainly focused on the characteristics of the procedure itself, without considering the specific implication this procedure could have for each patient [29].

It should be underlined that other items were also found to be under non-compliance with the informed consent form, which could have great ethical as well as legal ramifications; fundamentally, the civil liability derived from the breach of the duty to inform the patient in a personalized manner [12,30]: the lack of the complete identification of the patient, the lack of complete identification of the physician, and the signature of the physician. These deficiencies could be solved if the informed consent forms were created digitally from a digitized medical history and their completion by hand were not necessary [23].

The fact that anesthesia and critical care showed the greatest compliance with the identification of the physician and personalized risks could be due to the informed consent form information coming directly from the medical history in many cases. In contrast, the other disciplines obtained the information manually, and were therefore more susceptible to partial or complete forgetfulness regarding the inclusion of all criteria [13]. Also, the physician’s signature, which reveals the intention of the health professional, needs to be provided manually; as observed in this study, some disciplines, such as anesthesia and critical care, and gynecology, were more inclined to sign the forms.

Several studies indicate that the lack of awareness of healthcare professionals of their exact role in the process of obtaining informed consent may be behind the various errors that are made. As proposals for improvement, these studies suggest the development of protocols and joint teamwork between nurses and physicians, including nursing leadership, in obtaining informed consent [1,21,22], which has been shown to be effective and manageable [31]. Ensuring the patient’s correct understanding of the need for an intervention and its risks is another key element in ensuring patient autonomy, so protocols should include confirmation of the patient’s understanding and proactive involvement in decision-making [32]. In this process, nurses can be a key player due to their closeness to the patient and relatives. Nurses can help to bridge the gap in the patient-doctor communication process, not only by the helping relationship established but also by bringing biomedical terminology closer to the patient’s understandable language [21].

The main limitation of this study derives from the fact that it is a representative study of a local area, so its external validity could be limited. Although having analyzed the standardized informed consent documents by different scientific societies, used in most hospitals, the results could be generalized to the entire Spanish territory. On the other hand, the formal analysis of informed consents provides limited information to the document itself. Therefore, it is not possible to analyze whether the information provided to patients was adequate, sufficiently comprehensive or whether this information helped in decision-making. To resolve this aspect, it would be necessary to propose qualitative studies with in-depth interviews to help us understand the patients’ experiences during the process and to clarify the usefulness of the process for the promotion of autonomous decision-making.

## 5. Conclusions

The informed consent forms in critical care and surgical areas were shown to have a deficient formal quality due to the lack of compliance with important criteria such as personalized risks, contraindications, or providing a copy to the patient.

The compliance with these documents was inadequate, especially for the personalized risks and the complete identification of the patient and the physician.

The deficiencies in the formal quality and the compliance with the informed consent forms should be rectified to improve the information received by the patient to guarantee their freedom to choose and independence.

Protocols should be created for obtaining informed consent, with each professional’s role clearly described. The patient should be considered as a proactive element in decision-making. Due to the complexity of the continuous process of information and consent to interventions and decisions made about the patient, consideration should be given to the possibility of nursing professionals taking the lead in obtaining informed consent.

## Figures and Tables

**Table 1:** Formal quality criteria of the informed consent form.

#	Criteria
1	The hospital name must be shown
2	The service or unit where the IC form is being used must be identified
3	Spaces must be provided for the informing physician’s name, last names, registration number, and signature
4	Spaces must be provided for the name, last name, identification number/social security number/medical history number, and signature, of the patient who will be undergoing the procedure
5	Spaces must be provided for the name, last name, identification number, and signature, of the legal or family representative, or the person who represents the patient
6	Spaces must be provided for the date and place where the informed consent form was signed
7	The name of the procedure must be clearly identified
8	The nature and description of the procedure to be performed must be indicated
9	The aim/objective of the procedure must be indicated
10	The relevant or important contraindications must be shown
11	The probable risks and the typical risks under normal conditions must be indicated
12	Spaces must be provided for including important personalized risks
13	The contraindications must be present
14	Alternatives to the procedure must be present
15	The declaration of the patient of having adequately understood the information, and having all doubts clarified must the indicated
16	The declaration which states that consent can be revoked at any time, without indicating the cause for this, must be present.
17	A space must be provided for revoking the consent in case the patient considers it necessary
18	The indication by the patient or the legal representative which states that the patient provides consent for the procedure, must be present
19	It must be indicated in the document that the patient has been provided with a copy of it

Note: Adapted from Calle-Urra et al. [9].

**Table 2:** Form for assessing the compliance with the informed consent forms.

Items
Compliance with the physician’s name, last names, and registration Signature of the informing physician
Compliance with the name, last names, identification number/Social Security number/medical history number Signature of the patient who will be subjected to the procedure, if applicable
Contains the signature of the patient or legal representative. In the case that the legal representative signed, his or her name, last names and identification number must be included.
Contains the date and place where the form was signed
Includes the important personalized risks
Discipline to which the informed consent form belongs to

**Table 3:** Formal quality of the surgical consent forms of the hospital.

Discipline	Number of Consents	Non-Complied Formal Quality Criteria	Formal Quality Score
Anesthesia and Critical Care	6	13, 19	17
General surgery	67	3, 12, 13, 19	15
Dermatology	2	3, 5, 12, 13, 19	14
Gynecology	4	19	18
Ophthalmology	26	3, 5, 13, 18, 19	14
Otorhinolaryngology	39	2, 3, 16, 19	15
Traumatology	47	13, 19	17
Urology	33	2, 12, 13, 16, 19	14

**Table 4:** Surgical consent forms of the intervened patients.

Discipline	Frequency	Percentage
Anesthesia and Critical Care	188	50
General surgery	34	9
Dermatology	5	1.3
Gynecology	4	1.1
Ophthalmology	45	12
Otorhinolaryngology	15	4
Traumatology	61	16.2
Urology	24	6.4

**Table 5:** Percentage of compliance with the surgical consent forms from the hospital.

Item	Percentage
Date and place	96.8
Complete identification of the patient	93.9
Personalized risks	45.5
Complete identification of the physician	85.4
Physician’s signature	91.8
Patient’s signature	100
Complete identification of the representative	99.5
Signature of the representative	100

**Table 6:** Percentage of compliance with the most significant items.

Discipline	Physician Identification	Physician Signature	Personalized Risks
Anesthesia and Critical Care	100	100	84
General Surgery	61.8	76.5	11.8
Dermatology	60	80	20
Gynecology	75	75	25
Ophthalmology	73.3	82.2	15.6
Otorhinolaryngology	93.9	93.3	13.3
Traumatology	60.7	91.8	9.8
Urology	87.5	58.3	12.5

## Data Availability

The data used to support the findings of this study are available from the corresponding author upon request.
